# Prior event rate ratio adjustment produced estimates consistent with randomized trial: a diabetes case study

**DOI:** 10.1016/j.jclinepi.2020.03.007

**Published:** 2020-06

**Authors:** Lauren R. Rodgers, John M. Dennis, Beverley M. Shields, Luke Mounce, Ian Fisher, Andrew T. Hattersley, William E. Henley

**Affiliations:** a Institute of Health Research, University of Exeter Medical School, Exeter, UK; b NIHR Exeter Clinical Research Facility, University of Exeter Medical School, Exeter, UK; c IQVIA, London UK; d Department of Diabetes and Endocrinology, Royal Devon and Exeter NHS Foundation Trust, Exeter, UK

**Keywords:** PERR Pairwise, Electronic health record, Unmeasured confounding, Side-effects, Observational data, Pharmacovigilance

## Abstract

**Objectives:**

Electronic health records (EHR) provide a valuable resource for assessing drug side-effects, but treatments are not randomly allocated in routine care creating the potential for bias. We conduct a case study using the Prior Event Rate Ratio (PERR) Pairwise method to reduce unmeasured confounding bias in side-effect estimates for two second-line therapies for type 2 diabetes, thiazolidinediones, and sulfonylureas.

**Study Design and Settings:**

Primary care data were extracted from the Clinical Practice Research Datalink (*n* = 41,871). We utilized outcomes from the period when patients took first-line metformin to adjust for unmeasured confounding. Estimates for known side-effects and a negative control outcome were compared with the A Diabetes Outcome Progression Trial (ADOPT) trial (*n* = 2,545).

**Results:**

When on metformin, patients later prescribed thiazolidinediones had greater risks of edema, HR 95% CI 1.38 (1.13, 1.68) and gastrointestinal side-effects (GI) 1.47 (1.28, 1.68), suggesting the presence of unmeasured confounding. Conventional Cox regression overestimated the risk of edema on thiazolidinediones and identified a false association with GI. The PERR Pairwise estimates were consistent with ADOPT: 1.43 (1.10, 1.83) vs. 1.39 (1.04, 1.86), respectively, for edema, and 0.91 (0.79, 1.05) vs. 0.94 (0.80, 1.10) for GI.

**Conclusion:**

The PERR Pairwise approach offers potential for enhancing postmarketing surveillance of side-effects from EHRs but requires careful consideration of assumptions.

**What is new?**

**Key findings**
• Conventional analyses of side-effects based on adjustment for available confounders in electronic health record data gave results that were inconsistent with randomized trial estimates for one known side-effect of thiazolidinediones (edema) and one negative control outcome (gastrointestinal).
• Compared with conventional estimates, application of the Prior Event Rate Ratio (PERR) Pairwise method yielded estimates of side-effect risk that were more consistent with those observed in a large randomized trial of the same therapies.

**What this adds to what was known?**
• This case study provides the first substantive application of the PERR Pairwise method and shows how it can reduce unmeasured confounding in side-effect studies using EHR data; user-friendly R code to apply the PERR Pairwise method is provided.

**What is the implication and what should change now?**
• Unmeasured confounding is a threat to the validity of observational studies of drug side-effects.
• Researchers conducting pharmacovigilance studies using EHR data should consider the application of the PERR Pairwise approach to detect and adjust for unmeasured confounding.

## 1 Introduction

Postmarketing surveillance of new drugs (pharmacovigilance) is vital to ensure that patients receive safe and effective treatments. Longitudinal data from electronic health record (EHR) systems, such as the Clinical Practice Research Datalink (CPRD) in the UK, provide an increasingly important data source to study therapy effectiveness and risk of side-effects. A major challenge in utilizing observational data; however, is that patients are not randomly allocated to treatment as in a randomized controlled trial (RCT). For example, confounding by indication can arise where doctors preferentially prescribe one medication over another based on indication, severity, or prognosis [[1], [2], [3]]. A confounding variable (or confounder) is a variable that is related to both the allocation of treatment and the outcome (e.g., high BMI is associated with both statin prescribing and risk of cardiovascular events [4]). In practice, allocation to treatment may be subject to an unrecognized or unmeasured process (unmeasured confounding). Comparisons of treatment effects will be biased when one or more confounders is unmeasured, and this bias cannot be removed using standard analytical approaches.

Overcoming unmeasured confounding is a key challenge when drawing inferences from EHRs, and a growing number of approaches have been developed to address this. Historically, the principal approaches were instrumental variable (IV) analysis [[5], [6], [7]] and difference-in-differences [8]. Recent developments include the missing cause approach [9], regression discontinuity designs [10] and propensity score calibration [11,12]. Each of these methods relies on specific assumptions and can only be used for selected data structures. Comprehensive reviews are found in the study by Uddin et al., Streeter et al., and Alemayehu et al [[13], [14], [15]]. This study demonstrates the application of another promising approach to address unmeasured confounding in nonrandomized studies, the Prior Event Rate Ratio (PERR) method [[16], [17], [18]]. PERR is designed to reduce bias when comparing time-to-event or count outcomes between treatments, after the initiation of a new treatment. It is an extension of conventional regression-based approaches that exploits a before-and-after design to remove the effect of unmeasured confounders.

The conventional approach involves comparing outcome event rates after treatment initiation in individuals prescribed the treatment of interest (exposed group) compared with individuals prescribed a comparator treatment (unexposed group). Adjustment for baseline measured confounders is typically made using Cox proportional hazards (PH) regression. Differences between groups are summarized as a hazard ratio (HR_s_, where s denotes the study period, i.e., follow-up from the initiation of treatment). In EHR databases, information may also be available on the outcomes of interest before the initiation of the treatment (i.e., before the study baseline). A simple approach to accounting for group differences in prior outcomes [19] could add a binary indicator for a previous event in a study period model; however, this would not account for unobservable characteristics. PERR methodology extends this approach by incorporating differences in event rates between exposed and unexposed groups in the period before treatment initiation in the statistical model. The difference in event rates is represented as HR_p_. Because neither group receives the treatment of interest during the prior period, HR_p,_ with certain assumptions, reflects the influence of unmeasured confounders independent of treatment. The PERR adjusted hazard ratio, HR_PERR_, is calculated as HR_s_/HR_p_, and provides an estimate of the treatment effect adjusted for both measured and unmeasured confounders. The method requires the effect of the confounders (measured and unmeasured) to remain the same between periods (time-invariant unmeasured confounding) and treatment allocation for the study period not to be influenced by the outcome in the prior period (treatment decision to be independent of prior events) [17,18,20,21]. It is important to note that PERR is only applicable for nonterminal events which can reoccur in both periods; Appendix A has further details of PERR and relevant assumptions for its use.

Simulation studies [4,12,17] have explored the performance of the PERR method in a range of scenarios and demonstrated that it can produce biased treatment effect estimates when there is a relationship between prior outcomes and treatment selection or changes in the confounder effect between periods (time-varying unmeasured confounding). The original PERR approach is based on fitting two models which separate patient response in each period; recent work has developed an alternative formulation that keeps these data paired, using within-person comparisons to address the effects of time-invariant confounding. The PERR method was also shown in some cases to produce attenuated treatment effect estimates [17,18]; this bias is a consequence of the nonlinearity of the Cox model. PERR-ALT [17] is an alternative formulation using paired Cox regression which overcomes these issues. In PERR-ALT, HR_E_ is the HR comparing event rates in the study vs. prior period in the exposed group, and HR_U_ is the same comparison in the unexposed. HR_PERR-ALT_ is calculated as HR_E_/HR_U_. An extension to this methodology, PERR Pairwise [18], is based on the pairwise likelihood formulation of PERR-ALT.

Here, we set out to provide the first practical application of the PERR Pairwise method. As a case study, we aimed to apply PERR methodology to estimate risk of known side-effects from EHR data for medications commonly prescribed to lower blood glucose in type 2 diabetes (T2D), sulfonylureas (SUs), and thiazolidinediones (TZDs), and to compare the results with estimates from trial data in which participants were randomized to treatment.

## 2 Methods

### 2.1 Prior event rate ratio

Conventional analyses utilize data on confounders measured at the baseline and may include adjustment for prior events. PERR methodology adds an extra stage by selection of patients who have data available before initiation of new treatment and modeling of outcomes during this prior period (Appendix B). The time between starting the prior observation period and starting the study period is a parameter which should be chosen with care; a ratio method requires that the influence of the unmeasured confounders remain the same in the prior and study periods. The gap needs to be short enough to maintain the assumption of time-invariant confounding while long enough to ensure sufficient numbers of prior events are captured [17,18,21].

**Fig. 1:**
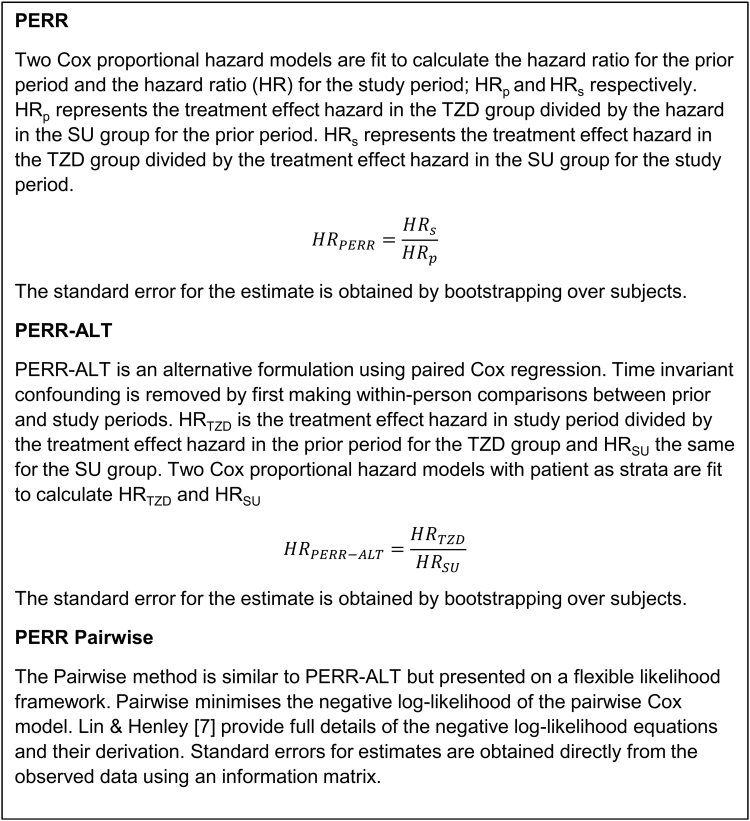
How to compute the PERR, PERR-ALT, and Pairwise estimates. *Abbreviations:* PERR, Prior Event Rate Ratio.

There are three formulations of PERR [[16], [17], [18]], summarized in Fig. 1 and Appendix A. We provide a worked example to enable other researchers to replicate this method easily. R code illustrating the computations for each method is provided (Supplementary 2). PERR-ALT and the newer Pairwise method reduce the bias inherent in the original PERR approach [17,18] and can be considered equivalent as both use paired Cox regression. The standard errors (SE) from PERR-ALT/Pairwise will often be larger than that of PERR [18] as patients only contribute if they have an event; for rare events, these formulations are less computationally stable than PERR. A computational limitation of PERR and PERR-ALT is that bootstrapping is required for SE; Pairwise is computed directly from the likelihood and produces direct estimates of SE. Given the computational advantages of using PERR Pairwise when faced with large sample sizes in EHR databases, we report Pairwise estimates as our selected within-subject approach with a note that PERR-ALT is an alternative. The PERR Pairwise approach also provides a natural framework for extending the method to address additional sources of complexity. For example, the underlying Pairwise model has a flexible period effect term which allows the proportional baseline hazards assumption to be relaxed in cases where the assumption is not met ([18]; Appendix A).

Our approach is first to investigate the influence of confounding using separate adjusted Cox PH models fitted to each period. Estimates of group differences in the prior period reflect differences in populations before the treatment was started and provide a measure of the degree of unmeasured confounding. An HR 95% confidence interval (CI) which does not contain 1 indicates a potential difference between the groups before allocation to the new treatment. However, even if 1 is contained within this interval, we recommend that the PERR method should still be applied [17]; only if PERR estimates are comparable with the study period Cox models that we can conclude no evidence of unmeasured confounding.

### 2.2 Case study: side-effects on type 2 diabetes medication

**Fig. 2:**
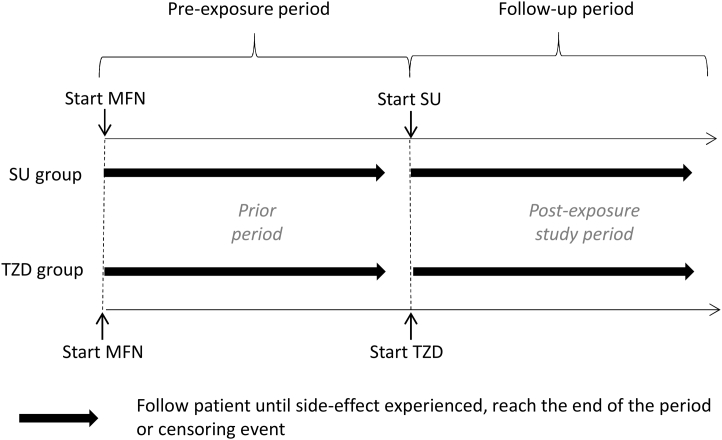
Schematic of Prior Event Rate Ratio method for our case study. SU group are equivalent to the “Unexposed” group and TZD to the “exposed” group in the standard definitions of the PERR methodology. Time between start MFN and start SU/TZD is a maximum of 5 y. Pre-exposure and follow-up periods are up to 2 y. *Abbreviations:* SU, sulfonylurea; TZD, thiazolidinedione; MFN, metformin; PERR, Prior Event Rate Ratio.

Patients with T2D are prescribed metformin (MFN) as first-line therapy; we use the experience of time on MFN as the period before the start of treatment [22]. Two common second-line treatments are TZD and SU. Known side-effects to TZDs are peripheral edema [23] and weight gain [24]; obtaining valid estimates of the rates of these in EHRs requires consideration of sources of confounding. We apply PERR Pairwise to assess and reduce the effect of any unmeasured confounding. In applications of PERR, we define an exposed and unexposed group; here, the TZD group is the exposed equivalent and SU the unexposed (Fig. 2). Gastrointestinal side-effects (GI) are not known to be associated with TZDs and are used as a negative control outcome (NCO) to test the robustness of the method. STROBE [25] guidelines were used to ensure transparency in our approach (Supplementary 1).

Patients with T2D were extracted on August 8, 2016 from CPRD [26]. The average year starting MFN was 2004 (Table 1). Patients who were prescribed MFN as their first-line treatment followed by SU or TZD were selected for the study; Fig. 2. The closest run of MFN treatment to the SU/TZD was used, followed by the first instance of SU/TZD. Two-year follow-up was used in each of the periods with the time recorded until patients experienced a side-effect, stopped/added to current treatment, died, or left the practice. 41,871 patients met our study criteria (32,242 on SU, 9,629 on TZDs; Appendix C).

**Table 1:** Descriptive statistics

Group	Gender (female)	Weight (kg)	BMI	Hba1c (mmol/mol [%])	Age (y)	Duration of diabetes (y)	Adherence %	Calendar year of start	Time from MFN start (mo)
SU Group (*N* = 32,242)									
Diagnosis	41%	95.0 (19.9) 15,404	33.1 (6.3) 15,384	75.8 (25.2) [9.1 (2.3)] 12,543	58.2 (11.0)				
MFN period		93.3 (19.7) 25,681	32.5 (6.2) 25,637	77.7 (21.1) [9.3 (1.9)] 23,034	59.9 (11.2)	1.7 (2.2)	87.9 (22.3) 25,917	2,005.2 (5.0)	
SU period		91.8 (19.7) 29,009	32.1 (6.1) 28,951	74.1 (17.6) [8.9 (1.6)] 28,423	61.7 (11.3)	3.5 (2.5)	89.7 (24.3) 23,039	2,007.1 (5.0)	19 [9–60]
TZD group (*N* = 9,629)									
Diagnosis	38.8%	98.3 (20.5) 4,605	34 (6.5) 4,603	78.3 (24.2) [9.3 (2.2)] 3,645	56.2 (10.3)				
MFN period		96.6 (20.1) 7,887	33.4 (6.4) 7,880	77.9(20.1) [9.3 (1.8)] 7,088	57.6 (10.4)	1.4 (1.9)	90.1 (20.3) 8,248	2,004.1 (2.8)	
TZD period		95.7 (20.1) 9,135	33.2 (6.3) 9,124	71.3 (14.8) [8.7 (1.4)] 9,123	59.4 (10.5)	3.2 (2.4)	92.5 (21.1) 8,205	2,005.9 (2.6)	18 [8–60]
ADOPT									
Diagnosis					SU 55.7 (10.1) TZD 55.5 (9.9)				
SU (*N* = 1,258)	41.4		32.2 (6.3)	56.9 (10.1) [7.4 (0.9)]	56.5 (10.2)	0.8 (0.9)			
TZD (*N* = 1,287)	44.5		32.2 (6.4)	57.0 (10.1) [7.4 (0.9)]	56.3 (10.0)	0.8 (0.9)			

*Abbreviations:* MFN, metformin; SU, sulfonylurea; TZD, thiazolidinedione; ADOPT, A Diabetes Outcome Progression Trial.

Mean (SD), median (IQR), or %. N reported where there are missing data.

An important consideration for setting up the study is the definition of periods; unmeasured factors influencing an individual's propensity for a side-effect independent of treatment should stay the same in both periods. To ensure this was a reasonable assumption, we restricted time between MFN and starting TZD/SU to 5 years; clinically, we would expect to review a patient's treatment regime within this period. We tested the sensitivity of our analysis to this period by restricting this to 3, 4, and 5 years [17], with little difference in results (Appendix D).

Side-effect outcomes were edema, GI, and weight gain. Medical codes for edema or GI identified the side-effects. Weight gain was calculated as change from the baseline [26]. Changes>20 kg were considered data errors and excluded. Significant weight gain was an increase from the baseline of 6% (5 kg increase in average baseline weight). Two consecutive weights were required to ensure that the weight gain was not temporary; the first falling within the 2 years from treatment start and the second between the first and up to 2 years after the end of the period.

Cox PH models, adjusted for measured covariates, were fitted to both periods. Unadjusted results are in Appendix E, and details of adjusted models in Appendix F. Covariates considered were baseline HbA1c, weight, BMI, age, duration of diabetes, gender, calendar year at the start of treatment, and adherence to medication [27]. Variables significant at the 10% level or with >15% influence on other coefficients were retained in multivariate models. All covariates were measured at the start of each period [26]. Where the use of covariates changed the sample size, we refit unadjusted models to the reduced sample to check whether results remained the same and to help diagnose any bias due to missing data. SQLyog and R version 3.3.1 were used.

### 2.3 Comparisons with other data/methods

To evaluate the validity of PERR results, we made comparisons with findings of the A Diabetes Outcome Progression Trial (ADOPT) study [28]. ADOPT was a multicentre, double-blind RCT evaluating the durability of glycemic control on TZD (rosiglitazone, *N* = 1,287), MFN, and SU (glibenclamide, *N* = 1,258). Patients were newly diagnosed (within 3 years) with T2D and had not been previously prescribed T2D medication. ADOPT was suitable for comparison as it compares the drugs of interest over a long period (4 years), the primary outcome was time to treatment failure and treatments were monitored for safety and tolerability. Side effects were well recorded.

We also compare PERR with an alternative method of adjusting for events in the prior period: a Cox PH model fitted to the data in the study period alone with an additional binary adjustment for whether the side-effect was experienced in the prior period (Prior Adjusted Study Model, PASM).

## 3 Results

Some differences in characteristics between patients who went on to TZDs as a second-line therapy compared with those who went on to SU were seen (Table 1); patients who were heavier and diagnosed younger tended to be prescribed TZDs rather than SU as second-line therapy (mean (SD): 93 (20) kg SU, 98 (21) kg TZD and 58 (11) yrs SU 56 (10) yrs TZD.) Minor differences in HbA1c and BMI were also seen at diagnosis. These may imply some prescriber bias. Both groups moved on to second-line therapy on similar time scales. ADOPT study patients were newly diagnosed and have a shorter duration of diabetes than the EHR patients; BMI and HbA1c at diagnosis are also lower.

### 3.1 Example 1: edema

**Fig. 3:**
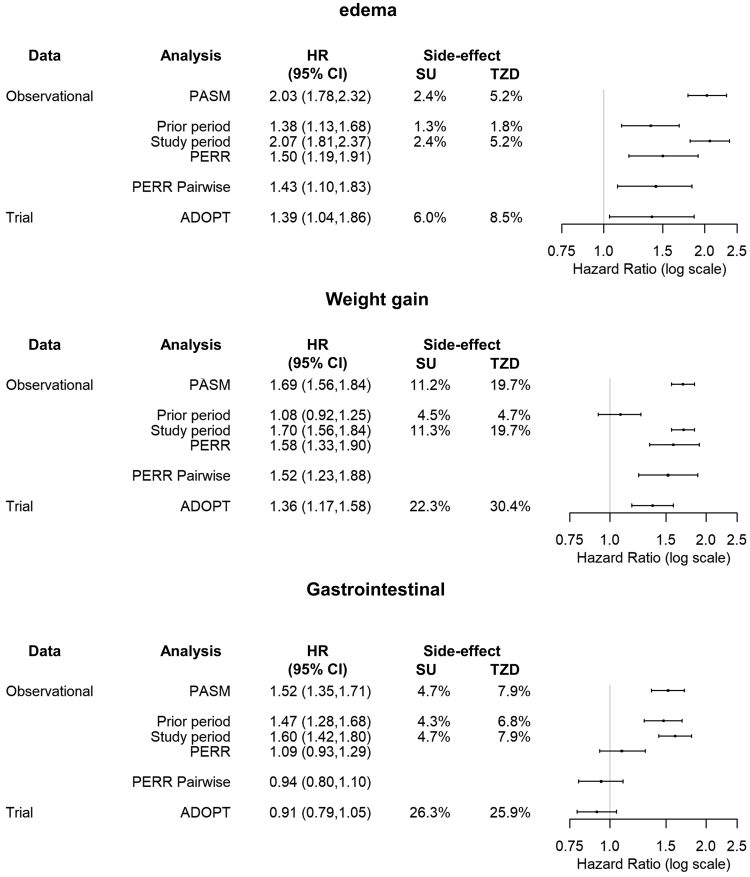
Case study results: adjusted models, PERR Pairwise *N* = 32,242 SU *N* = 9,629 TZD in edema and gastrointestinal analyses, *N* = 10,807 SU *N* = 4,468 weight gain analysis. Column 4 shows percentage of patients who experience the side-effect in each period. An HR greater than 1 indicates a greater risk of side-effect in the TZD group relative to SU. An HR less than 1 indicates greater risk of a side-effect in the SU group. HR from the prior and study are used to calculate PERR; PERR Pairwise is calculated by minimizing the negative log-likelihood of the paired Cox model. *Abbreviations:* SU, sulfonylurea; TZD, thiazolidinedione; MFN, metformin; HR, hazard ratio; PERR, Prior Event Rate Ratio.

The rate of edema on MFN was higher in the TZD group, 1.8% (*n* = 177), than the SU group, 1.3% (*n* = 427). Increased difference in risk of edema was observed in the study period, with risks of 5.2% (*n* = 498) vs. 2.4% (*n* = 767) for TZD and SU groups, respectively. The incidence of edema was higher in the TZD group than the SU group in the prior period after adjustment for measured confounders, HR 95% CI 1.39 (1.17, 1.66), Fig. 3. This difference suggests unmeasured confounding; the group that later was prescribed TZDs were more prone to edema. The study model indicates the risk of edema is more than doubled in the TZD group, 2.07 (1.81, 2.37) with a similar estimate in the PASM, 2.03 (1.78, 2.32). ADOPT showed a greater risk of edema on TZD but a smaller effect; 1.39 (1.04, 1.86). The PERR Pairwise analysis also showed a greater risk of edema with TZDs but results more closely replicated ADOPT, 1.43 (1.10, 1.83), than PASM and the conventional Cox PH model. The standard PERR analysis gave similar results to PERR Pairwise.

### 3.2 Example 2: weight gain

Patients had similar weight gain on MFN in both groups (TZD: 5.8%, *n* = 1,126; SU: 6.2%, *n* = 356), but greater gain when on TZD (23.8%, *n* = 1.477) than SU (13.2%, *n* = 2.581). Comparison of adjusted models indicated little influence of unmeasured confounding from group differences in the prior (Fig. 3): Cox study model, 1.70 (1.56, 1.84), and Pairwise, 1.52 (1.23, 1.88). The PASM and PERR showed similar results, 1.69 (1.56, 1.84) and 1.58 (1.33, 1.90). EHR analyses found a greater risk of weight gain for patients taking TZD relative to SU; the CIs overlapped the ADOPT trial, 1.36 (1.17, 1.58).

### 3.3 Example 3: gastrointestinal side-effects

Patients in the TZD group experienced more GI in both periods, 9.8% (*n* = 945) and 10.2% (*n* = 980), than those in the SU group, 6.0% (*n* = 1,948) and 6.4% (*n* = 2,053). In adjusted analyses, the CI in the prior period (1.28, 1.68; Fig. 3), suggested unmeasured confounding. However, after adjustment for unmeasured confounding using PERR Pairwise, there was no association between GI and TZD, 0.94 (0.80, 1.10), consistent with ADOPT, 0.91 (0.79, 1.05). PERR produced a similar estimate, 1.09 (0.93, 1.29). The PASM reduced the HR closer to that of ADOPT than the study model alone, 1.52 (1.35, 1.71) vs. 1.60 (1.42, 1.80) but did not remove the false association between GI and TZD.

## 4 Discussion

We showed unmeasured confounding affected the analysis of side-effects of T2D medications using EHR data. When adjustment for confounding using PERR Pairwise was applied to edema, it yielded estimates consistent with RCT data. In the NCO example, Pairwise eliminated the false association between TZD and GI. Comparing the PASM to the study model, the PASM did reduce the HRs. However, PERR Pairwise gave estimates that were much closer to the trial results than PASM. PASM did not eliminate the false association between TZD and GI. For weight gain, there was no evidence of unmeasured confounding; and, while the HR from the study period alone did overlap the trial, the Pairwise estimate was closer to the ADOPT result.

This case study illustrates how PERR methodology is a potentially useful approach to addressing unmeasured confounding in evaluation studies based on real-world data. Applications of this methodology to date have tended to use PERR rather than PERR Pairwise. The original PERR formulation estimates were closer to the clinical trial results than the study model alone. However, the PERR estimates were consistently further from the trial results than the Pairwise method and it is known that PERR does not completely remove bias resulting from the nonlinearity of the Cox model [18]. Pairwise produces larger SE ([18]; Appendix A) but this is unlikely to be a substantial problem with EHR sample sizes. We advocate use of PERR Pairwise as an additional component of the tool kit for applied researchers tackling the issue of unmeasured confounding; only if this concurs with the adjusted Cox study model can we conclude no evidence of unmeasured confounding.

As with all adjustment methods for unmeasured confounding, the PERR approach requires certain assumptions to be met to provide valid estimates. PERR-ALT/Pairwise can produce unbiased estimates when the unmeasured confounding is time-invariant, but simulations showed that time-dependent confounding is a potentially important source of bias when applying the PERR approach [4,17,18]; it cannot be removed from the data using available methods. The results of any study utilizing PERR, including this one, are only valid under this assumption. We addressed this issue by careful consideration of the time between the start of the prior and start of the study period with sensitivity analysis using shorter periods. Where possible, the choice of a prior period should minimize the risk of time-dependent confounding (e.g., limiting the time between periods).

Sample size may also be an issue when adjusting for baseline covariates, some of which are poorly recorded. A limitation of EHR data is that reporting of outcomes may be incomplete and subject to error. Weight, in particular, was not well reported, and there is evidence of a relationship between weight recorded and weight change [29,30]; moreover, interval-censored data and imprecise measuring times within our weight data could bias results toward the null [31]. Both periods should be affected similarly, but further work may be required to investigate a complex picture of weight gain. Another assumption is that prior events do not influence the likelihood of future treatment. Previous work [4,18,20,21] showed bias in PERR when prior events influence treatment selection. Although this can be an issue in effectiveness studies, it is less likely to be problematic when modeling side-effects, under the assumption that treatments are not allocated according to the potential (and possibly unknown) side-effects of the treatment. However, further work is required to assess the validity of this assumption before PERR is recommended for widespread use in pharmacovigilance studies.

Alternative approaches to unmeasured confounding include IV analysis [[5], [6], [7]], regression discontinuity designs [10], missing cause [9] and propensity score regression calibration [12]. To date, there have been no studies which have compared the relative merits of these methods with PERR. There is no single solution to the problem of unmeasured confounding; exploring which methods perform best under different conditions via clinically informed simulations and case studies is necessary. The choice of method will always depend on the particular case at hand; for example, PERR inappropriate for terminal outcomes, IV studies require a suitable instrument. With increasing volume of EHR data, there is a need to continue developing these methods and to utilize these data as efficiently as possible. One particular challenge and important topic for future research is the need to identify strategies for addressing time-dependent unmeasured confounding.

## 5 Conclusions

This article illustrates how to apply the PERR Pairwise method in detecting and adjusting for unmeasured confounding when assessing risk of side-effects from EHR data. It is relatively straightforward to implement and can be used to provide “real-world” estimates of risk for both known and emerging side-effects in pharmacovigilance studies where trials are not available. The approach requires strong assumptions and further work is needed to provide guidance on addressing time-dependent confounding.

## CRediT authorship contribution statement

**Lauren R. Rodgers:** Conceptualization, Methodology, Formal analysis, Writing - original draft, Writing - review & editing. **John M. Dennis:** Validation, Writing - review & editing. **Beverley M. Shields:** Investigation, Writing - review & editing. **Luke Mounce:** Conceptualization, Writing - review & editing. **Ian Fisher:** Conceptualization, Funding acquisition, Writing - review & editing. **Andrew T. Hattersley:** Conceptualization, Supervision, Funding acquisition, Writing - review & editing. **William E. Henley:** Conceptualization, Methodology, Funding acquisition, Writing - review & editing.

## Supplementary data

Data Profile
