# Supplementary material for: Prior event rate ratio adjustment produced estimates consistent with randomized trial: a diabetes case study
Source: J Clin Epidemiol. 2020 Jun;122:78–86. doi: 10.1016/j.jclinepi.2020.03.007 (PMC7262589; doi:10.1016/j.jclinepi.2020.03.007)
Supplement: Supplementary 1 [file mmc2.docx]

**Supplementary 1: The RECORD statement – checklist of items, extended from the STROBE statement, that should be reported in observational studies using routinely collected health data.**

|  | **Item No.** | **STROBE items** | **Location in manuscript where items are reported** | **RECORD items** | **Location in manuscript where items are reported** |
| --- | --- | --- | --- | --- | --- |
| **Title and abstract** | | | | | |
|  | 1 | (a) Indicate the study’s design with a commonly used term in the title or the abstract (b) Provide in the abstract an informative and balanced summary of what was done and what was found |  | RECORD 1.1: The type of data used should be specified in the title or abstract. When possible, the name of the databases used should be included.  RECORD 1.2: If applicable, the geographic region and timeframe within which the study took place should be reported in the title or abstract.  RECORD 1.3: If linkage between databases was conducted for the study, this should be clearly stated in the title or abstract. | Title and  Abstract para 2, pg 2  Abstract para 2 pg 2  NA |
| **Introduction** | | | | | |
| Background rationale | 2 | Explain the scientific background and rationale for the investigation being reported |  |  | Introduction para 1-3 pg 5-6 |
| Objectives | 3 | State specific objectives, including any prespecified hypotheses |  |  | Page 8 para 1  “utilising information prior to the start of new therapy can identify bias in side-effect estimates in patients prescribed different medications and how to remove this.” |
| **Methods** | | | | | |
| Study Design | 4 | Present key elements of study design early in the paper |  |  | Introduction para 3-5Prior event rate ratio section pg 8 for statistical methods and case study section pg 9 for specifics to example.  Figures 1 and 2 |
| Setting | 5 | Describe the setting, locations, and relevant dates, including periods of recruitment, exposure, follow-up, and data collection |  |  | Case study pg 9-12, further details published separately in protocol (reference 32) |
| Participants | 6 | *(a) Cohort study* - Give the eligibility criteria, and the sources and methods of selection of participants. Describe methods of follow-up  *Case-control study* - Give the eligibility criteria, and the sources and methods of case ascertainment and control selection. Give the rationale for the choice of cases and controls  *Cross-sectional study* - Give the eligibility criteria, and the sources and methods of selection of participants  *(b) Cohort study* - For matched studies, give matching criteria and number of exposed and unexposed  *Case-control study* - For matched studies, give matching criteria and the number of controls per case |  | RECORD 6.1: The methods of study population selection (such as codes or algorithms used to identify subjects) should be listed in detail. If this is not possible, an explanation should be provided.  RECORD 6.2: Any validation studies of the codes or algorithms used to select the population should be referenced. If validation was conducted for this study and not published elsewhere, detailed methods and results should be provided.  RECORD 6.3: If the study involved linkage of databases, consider use of a flow diagram or other graphical display to demonstrate the data linkage process, including the number of individuals with linked data at each stage. | Case study section pg 9-12, codes published on clinicalcodes.org, more detail in protocol published reference 32. Flow diagram Appendix B and C  Protocol published reference 32 plus details in case study section pg 9-12. Validation detailed in Validation of Method section pg 13, trial details reference 34  NA |
| Variables | 7 | Clearly define all outcomes, exposures, predictors, potential confounders, and effect modifiers. Give diagnostic criteria, if applicable. |  | RECORD 7.1: A complete list of codes and algorithms used to classify exposures, outcomes, confounders, and effect modifiers should be provided. If these cannot be reported, an explanation should be provided. | Medical codes published on clinicalcodes.org. Details of variables in case study section 3.2. Appendix F details covariates selected for final models. Method for covariate selection para 4 of case study pg 11. |
| Data sources/ measurement | 8 | For each variable of interest, give sources of data and details of methods of assessment (measurement).  Describe comparability of assessment methods if there is more than one group |  |  | Case study section para 3-4, pg 12, protocol published reference 32 and Appendix F |
| Bias | 9 | Describe any efforts to address potential sources of bias |  |  | Description of method in prior event rate ratio method in introduction, methods section 2 and outcome of case study, Figures 1-3 |
| Study size | 10 | Explain how the study size was arrived at |  |  | Protocol published reference 32 plus case study details section 2.2 |
| Quantitative variables | 11 | Explain how quantitative variables were handled in the analyses. If applicable, describe which groupings were chosen, and why |  |  | Case study details para 3-4 pg 11, model selection details pg 12, details of final models Appendix F, descriptive statistics in Table 1 |
| Statistical methods | 12 | (a) Describe all statistical methods, including those used to control for confounding  (b) Describe any methods used to examine subgroups and interactions  (c) Explain how missing data were addressed  (d) *Cohort study* - If applicable, explain how loss to follow-up was addressed  *Case-control study* - If applicable, explain how matching of cases and controls was addressed  *Cross-sectional study* - If applicable, describe analytical methods taking account of sampling strategy  (e) Describe any sensitivity analyses |  |  | a) Prior event rate ratio section 2.1, Figures 1 and 2  b) NA  c) Protocol reference 32, pg 12  “Where the use of covariates changed the sample size we refit unadjusted models to the smaller data set to check that results remained the same.”  d) Case study details  e) Comparision to trial in Validation of Method 2.3 |
| Data access and cleaning methods |  | .. |  | RECORD 12.1: Authors should describe the extent to which the investigators had access to the database population used to create the study population.  RECORD 12.2: Authors should provide information on the data cleaning methods used in the study. | Reference 32, article contribution and data reporting statements pg 18statement  Case study section pg 11-12, reference 32, Appendix F |
| Linkage |  | .. |  | RECORD 12.3: State whether the study included person-level, institutional-level, or other data linkage across two or more databases. The methods of linkage and methods of linkage quality evaluation should be provided. | NA |
| **Results** | | | | | |
| Participants | 13 | (a) Report the numbers of individuals at each stage of the study (*e.g.*, numbers potentially eligible, examined for eligibility, confirmed eligible, included in the study, completing follow-up, and analysed)  (b) Give reasons for non-participation at each stage.  (c) Consider use of a flow diagram |  | RECORD 13.1: Describe in detail the selection of the persons included in the study (*i.e.,* study population selection) including filtering based on data quality, data availability and linkage. The selection of included persons can be described in the text and/or by means of the study flow diagram. | Case study details pg 10-12, protocol reference 32. Flow chart Appendix C. General method described in Appendix A, B. |
| Descriptive data | 14 | (a) Give characteristics of study participants (*e.g.*, demographic, clinical, social) and information on exposures and potential confounders  (b) Indicate the number of participants with missing data for each variable of interest  (c) *Cohort study* - summarise follow-up time (*e.g.*, average and total amount) |  |  | Results para 1 page 13-15. Table 1, Figure 3 |
| Outcome data | 15 | *Cohort study* - Report numbers of outcome events or summary measures over time  *Case-control study* - Report numbers in each exposure category, or summary measures of exposure  *Cross-sectional study* - Report numbers of outcome events or summary measures |  |  | Case study paragraph 1 pg 10, Figure 3, Table 1 and Appendix F |
| Main results | 16 | (a) Give unadjusted estimates and, if applicable, confounder-adjusted estimates and their precision (e.g., 95% confidence interval). Make clear which confounders were adjusted for and why they were included  (b) Report category boundaries when continuous variables were categorized  (c) If relevant, consider translating estimates of relative risk into absolute risk for a meaningful time period |  |  | Case study examples pg 12-15, Figure 3. Figure Appendix E for unadjusted results and further details of final models in Appendix F. |
| Other analyses | 17 | Report other analyses done—e.g., analyses of subgroups and interactions, and sensitivity analyses |  |  | Case study comparison to clinical trial; case study section pg 10-12. Figure 3 |
| **Discussion** | | | | | |
| Key results | 18 | Summarise key results with reference to study objectives |  |  | Discussion para 1 pg 15 |
| Limitations | 19 | Discuss limitations of the study, taking into account sources of potential bias or imprecision. Discuss both direction and magnitude of any potential bias |  | RECORD 19.1: Discuss the implications of using data that were not created or collected to answer the specific research question(s). Include discussion of misclassification bias, unmeasured confounding, missing data, and changing eligibility over time, as they pertain to the study being reported. | Discussion para 2 |
| Interpretation | 20 | Give a cautious overall interpretation of results considering objectives, limitations, multiplicity of analyses, results from similar studies, and other relevant evidence |  |  | Discussion para 2-3 |
| Generalisability | 21 | Discuss the generalisability (external validity) of the study results |  |  | Discussion para 4, conclusions  “The Pairwise method provides a potentially useful approach to detecting and adjusting for unmeasured confounding in pharmacovigilance studies where trials are not available.” |
| **Other Information** | | | | | |
| Funding | 22 | Give the source of funding and the role of the funders for the present study and, if applicable, for the original study on which the present article is based |  |  | Funding statement and conflicts of interest pg 17 |
| Accessibility of protocol, raw data, and programming code |  | .. |  | RECORD 22.1: Authors should provide information on how to access any supplemental information such as the study protocol, raw data, or programming code. | Data reporting statement pg 18 |

*Reference: Benchimol EI, Smeeth L, Guttmann A, Harron K, Moher D, Petersen I, Sørensen HT, von Elm E, Langan SM, the RECORD Working Committee. The REporting of studies Conducted using Observational Routinely-collected health Data (RECORD) Statement. *PLoS Medicine* 2015; in press.

*Checklist is protected under Creative Commons Attribution ([CC BY](http://creativecommons.org/licenses/by/4.0/)) license.
