# Supplementary material for: Prior event rate ratio adjustment produced estimates consistent with randomized trial: a diabetes case study
Source: J Clin Epidemiol. 2020 Jun;122:78–86. doi: 10.1016/j.jclinepi.2020.03.007 (PMC7262589; doi:10.1016/j.jclinepi.2020.03.007)
Supplement: Supplementary 2 [file mmc3.docx]

**Supplementary 2: R Code for the three methods**

Data set d with columns

cens_prior
cens_study
trt_prior
trt_study
time_prior
time_study
covar_prior
covar_study
patient_id

**PERR Method**require(survival)

#Prior period model

modelprior<-with(d,coxph(Surv(time_prior,cens_prior)~ trt_study
 +covar_prior))

#Study period model

modelstudy<-with(d,coxph(Surv(time_study,cens_study)~ trt_study
 +covar_study))

#PERR estimate

perr_estimate<- exp(as.numeric(modelstudy$coef[1])-
 as.numeric(modelprior$coef[1]))

#bootstrap for standard errors

bootn<-1000
perr_boot<-rep(0,bootn)

perr_se<- for (i in 1:bootn){
 d_boot <- d[sample(nrow(d),nrow(d),replace=T), ]
 perr_boot[i]<-with(d_boot,
 coxph(Surv (time_study,cens_study)~
 trt_study+covar_study)$coef)[1]-
 coxph(Surv(Surv(time_prior,cens_prior)~
 trt_study+covar_prior)$coef)[1]
 )
 }

#Order bootstrapped results

perr_se<-perr_se[order(perr_se),]

#95% CI for PERR estimate

perr_ci<-c(perr_se[25],perr_se[975])

**PERR-ALT**

require(survival)

supatients<-d$patid[d$drug_study==”SU”]
tzdpatients<-d$patid[d$drug_study==”TZD”]

d_perralt<-reshape(d,varying=c(‘cens_prior’,’cens_study’,
 ’time_prior’,’time_study’,’drug_prior’,
 ’drug_study’,’covar_prior’,’covar_study’),
 time=’period’,idvar=’patient_id’,
 direction=’long’,sep=’_’)

#SU group

modelsu<-with(subset(d_perralt,patient_id%in%supatients),
 coxph(Surv(time,cens)~ period+
 covar+strata(patient_id)))

#TZD group

modeltzd<-with(subset(d_perralt,patient_id%in%tzdpatients),
 coxph(Surv(time,cens)~ period+
 covar+strata(patient_id)))

# PERR-ALT Estimate

perralt<- exp(as.numeric(modeltzd$coef[1])-
 as.numeric(modelsu$coef[1]))

#Boot strap for standard error

bootn<-1000
perralt_boot<-rep(0,bootn)

perralt_se<- for (i in 1:bootn){
 dalt_boot <-d_perralt[sample(nrow(d_perralt),
 nrow(d_perralt),replace=T), ]
 perralt_boot[i]<-with(subset(dalt_boot,
 patient_id%in%supatients),
 coxph(Surv(time,cens)~ period +
 covar+strata(patient_id)))$coef)[1]-
 with(subset(dalt_boot,
 patient_id%in%tzdpatients),
 coxph(Surv(time,cens)~ period+
 covar+strata(patient_id)))coef)[1]

}

#Order boot strapped results

perralt_se <- perralt_se[order(perralt_se),]

#95% CI for PERR estimate

perralt_ci<-c(perralt_se[25],perralt_se[975])

**PERR Pairwise (adapted from Lin & Henley (5))**

# define likelihood functions

l<-function(theta_3, paitheta_2, theta_12){

sum(-d$dp*log(1+d$p*exp(theta_12*d$drug+paitheta_2+
 theta_3*(d$covar_study-d$covar_prior)))+
 d$ds*(theta_12*d$drug +paitheta_2+
 theta_3*(d$covar_study-d$covar_prior)-
 log(exp(theta_12*d$drug+paitheta_2+
 theta_3*(d$covar_study-d$covar_prior))+d$s)))
 }

lnlm<-function(p){-l(p[1],p[2],p[3])}

#Pairwise calculation

d$dp=ifelse(d$cens_prior==0,0,1)
d$ds=ifelse(d$cens_study==0,0,1)
d$p=ifelse(d$time_prior<=d$time_study,1,0)
d$s=ifelse(d$time_study<=d$time_prior,1,0)
d$drug=ifelse(d$drug_study=="TZD",1,0)

#minimise the negative log-likelihood

pairwise<-nlm(lnlm_none, c(0,0))

#extract parameter estimates

theta_3<-pairwise$estimate[1]
paitheta_2<-pairwise$estimate[2]
theta_12<-pairwise$estimate[3]

#Create information matrix and solve to find standard error

A<-with(d,exp(theta_12*drug+paitheta_2+
 theta_3*(covar_study-covar_prior))*
 (ds*s/(exp(theta_12*drug+paitheta_2+
 theta_3*( covar_study-covar_prior))+s)^2+
 dp*p/(1+p*exp(theta_12*drug+paitheta_2+
 theta_3*(covar_study-covar_prior)))^2)
 )

I<-rep(0,9)
dim(I)<-c(3,3)
I[1,1]<-with(d,sum(A*(covar_study-covar_prior)^2))
I[1,2]<-with(d,sum(A*(covar_study-covar_prior)))
I[2,1]<-I[1,2]
I[1,3]<-with(d,sum(A*(covar_study-covar_prior)*drug))
I[3,1]<-I[1,3]
I[2,2]<-sum(A)
I[2,3]<-with(d,sum(A*drug))
I[3,2]<-I[2,3]
I[3,3]<-with(d,sum(A*drug^2))

sd_error<-sqrt(solve(I)[3,3])

#PERR Pairwise Estimate

perrpair<-exp(theta_12)

#95% CI

perrpair_ci<-c(exp(theta_12-1.96*sd_error),
 exp(theta_12+1.96*sd_error))
