# Supplementary material for: Prior event rate ratio adjustment produced estimates consistent with randomized trial: a diabetes case study
Source: J Clin Epidemiol. 2020 Jun;122:78–86. doi: 10.1016/j.jclinepi.2020.03.007 (PMC7262589; doi:10.1016/j.jclinepi.2020.03.007)
Supplement: Appendix [file mmc4.docx]

**Appendix A: PERR Methodology**

The Prior Event Rate Ratio (PERR) method is a quasi-experimental approach to estimating treatment or exposure effects from non-randomised studies. A limitation of conventional regression methods is that they can only account for group imbalances that can be explained using measured confounding variables. The PERR methodology addresses the potential for unmeasured confounding by using a before-and-after design in which the outcomes of interest are modelled both before and after a change or addition to treatment. It is based on a regression modelling framework, using models for time-to-event or count data, and is similar to the Difference-in-Differences approach originally developed in econometrics (Streeter et al, 2017). The approach is suitable for repeatable events that can be defined for a period before and after the change or addition to treatment. The methodology is not applicable to one-off outcomes such as death.

We now outline the framework for PERR analysis and give details of different models that have been proposed. The general approach involves comparing event rates for the same (potentially repeatable) outcome in a prior period and a study period for two groups; the unexposed (U) and exposed groups (E). It is a requirement that neither group received the ‘new’ treatment in the prior period and only the exposed group received it at the start of the study period. The treatment estimate from the prior period is used to capture the degree of unmeasured confounding. Under relevant assumptions (see below), bias in the estimated effect of the new treatment (from the study period) is removed by correcting for the treatment estimate from the prior period, without the need to adjust for the unmeasured confounder(s).

**Model assumptions**

1. The event rate for the outcome of the exposed and unexposed groups before the start of the study period should reflect all confounders (measured and unmeasured) independent of treatment.
   This assumptions holds only if neither group has received the new treatment previously. The Hazard Ratio (HR) for the prior period should reflect the aggregated influence of the confounders, measured and unmeasured, Where there is no influence of unmeasured confounders the PERR result should be similar to the Cox Proportional HR(1).
2. The distribution and effects on the unmeasured confounders in both groups is the same in the prior and study periods. The influence of prior events within an individual can differ between exposed and unexposed groups, and the within-person specification of PERR-ALT and PERR Pairwise provides improved estimates compared with PERR alone. Under time-dependent confounding, where the distribution and effects of the unmeasured confounders changes over time, bias can arise in the treatment effect estimates. This has been shown to be the biggest influence on bias in simulation studies (2,3).
3. Outcome in the prior period does not influence the allocation of future treatment. Any association between prior events and whether or not a patient is subsequently prescribed treatment can lead to bias in the treatment effect estimates (3–5). Thought is required for the context in the application of the PERR methodology to determine in each case if PERR is appropriate.

**PERR Model**

We fit two Cox Proportional Hazards models to compare the unexposed and exposed groups, one for the outcome in the prior period and one for the outcome in the study period. The first model calculates the HR for the outcome in the prior period, HR_p,_ which is the hazard for the exposed group in the prior period divided by the hazard in the unexposed group in the prior period

$$HR_{p}=\frac{H_{E,p}}{H_{U,p}}$$

The second model calculates the HR for the outcome in the study period, HR_p,_ which is the hazard for the exposed group in the study period divided by the hazard in the unexposed group in the study period

$$HR_{s}=\frac{H_{E,s}}{H_{U,s}}$$

HR_PERR_ is defined as the ratio of these two

$$HR_{PERR}=\frac{HR_{s}}{HR_{p}}$$

The same subjects are in both the prior and study periods; a condition of a subject meeting the entry criteria for the analysis is to have information on a prior period and entry to the study period.

Bootstrapping is used to obtain standard errors for estimates. Bootstrapping is done by sampling individuals; thus in each bootstrapping iteration the same subjects are in the prior and study periods.

In simulation studies (13, 14), the PERR method was frequently shown to produce attenuated treatment effect estimates; this bias is a consequence of the non-linearity of the Cox model (14). The PERR-ALT and Pairwise formulations of the model overcome this problem and produce less biased results.

**PERR-ALT**

This formulation of PERR uses Paired Cox regression(2). Two Cox Proportional Hazards models are fit with subjects as strata. The first model calculates the HR for the outcome in the exposed group, HR_E,_ which is the hazard for the exposed group in the study period divided by the hazard in the prior period

$$HR_{E}=\frac{H_{E,S}}{H_{E,p}}$$

The second model calculates the HR for the outcome in the unexposed group, HR_U,_ which is the hazard for the unexposed group in the prior period divided by the hazard in the study period

$$HR_{U}=\frac{H_{U,S}}{H_{U,p}}$$

HR_PERR-ALT_ is defined as the ratio of these two

$$HR_{PERR-ALT}=\frac{HR_{E}}{HR_{U}}$$

The PERR method was found to produce attenuated treatment effect estimates in simulation studies(2), the within-person estimates of PERR-ALT gives unbiased estimates provided there is no time-dependent unmeasured confounder influence. Although, in rare outcomes the PERR estimates are more computationally stable(2).

Bootstrapping is used to obtain standard errors for PERR-ALT estimates. Bootstrapping is done by sampling individuals; thus in each bootstrapping iteration the same subjects are in the prior and study periods.

**PERR Pairwise**

The Pairwise PERR is an equivalent formulation of PERR-ALT. Where PERR-ALT is calculated using two paired Cox regression models Lin & Henley(3) have derives the pairwise Cox likelihood function to calculate the estimate. This approach is more flexible and quicker to produce estimates and standard errors. The standard errors are calculated directly from the data using an information matrix.

We define $T_{p}^{*}$ is the time to an event in the prior period and $T_{p}^{+}$ the prior censoring time. $\Delta_{p}$is an indicator variable which equals 1 if $T_{p}^{*}\leq T_{p}^{+}$ and 0 otherwise. With $\Delta_{s}$ defined similarly for the study period. Thus patients only contribute to the likelihood if they have an event. θ_12_ is the parameter of interest; the difference in effect between our two therapies θ_1_ and θ_2_, $\theta_{12}=\theta_{1}-\theta_{2}$. We set $X_{i1}=1$ if subject *i* is in theTZD group and 0 otherwise. . $X_{i3}$and $X_{i4}$ are measured covariates in the prior and study period respectively and $\theta_{3}$ the respective coefficient. $\alpha$ is a period effect, a time component of the model.

The log likelihood as defined in (3), which corresponds to the R code in the Supplementary material;

$$l_{full}(\theta_{3},\alpha+\theta_{2},\theta_{12}),=\sum_{i} \left( -\Delta_{p}\log\left( 1+pe^{\theta_{12}X_{i1}+\alpha+\theta_{2}+\theta_{3}\left( X_{i4}-X_{i3} \right)} \right)+\Delta_{s}\left( \theta_{12}X_{i1}+\alpha+\theta_{2}+\theta_{3}\left( X_{i4}-X_{i3} \right)-\log\left( e^{\theta_{12X_{i1}}+\alpha+\theta_{2}+\theta_{3}\left( X_{i4}-X_{i3} \right)}+s \right) \right) \right)$$

To calculate PERR Pairwise we minimise the log-likelihood function to obtain an estimate of $\theta_{1}-\theta_{2}$.

$$PERR Pairwise=e^{\hat{\theta}_{12}}$$

Subjects only contribute to the likelihood function if they have an event; this will reduce the sample size in calculations and is likely the reason that PERR-ALT, and hence PERR Pairwise, are less computationally stable in rare events and have larger confidence intervals than the original PERR (2) Pairwise is considered a flexible model as it can be extended for time varying covariates and the α period effect term specified (3).

1.

Tannen RL, Weiner MG, Xie DW. Use of primary care electronic medical record database in drug efficacy research on cardiovascular outcomes: comparison of database and randomised controlled trial findings. Br Med J. 2009;338.

2. Yu M, Xie D, Wang X, Weiner MG, Tannen RL. Prior event rate ratio adjustment: numerical studies of a statistical method to address unrecognized confounding in observational studies. Pharmacoepidemiol Drug Saf. 2012/05/11. 2012;21 Suppl 2:60–8.

3. Lin NX, Henley WE. Prior event rate ratio adjustment for hidden confounding in observational studies of treatment effectiveness: a pairwise Cox likelihood approach. Stat Med. Wiley-Blackwell; 2016 Dec 10;35(28):5149–69.

4. Gallagher A, De Vries F, Van Staa T. Prior event rate ratio adjustment: a magic bullet or more of the same? Pharmacoepidemiol Drug Saf. 2009;18(S14–S15).

5. Uddin MJ, Groenwold RHH, van Staa TP, de Boer A, Belitser S V, Hoes AW, et al. Performance of prior event rate ratio adjustment method in pharmacoepidemiology: a simulation study. Pharmacoepidemiol Drug Saf. 2015 May;24(5):468–77.

**B: Flowchart of dataset construction**

**
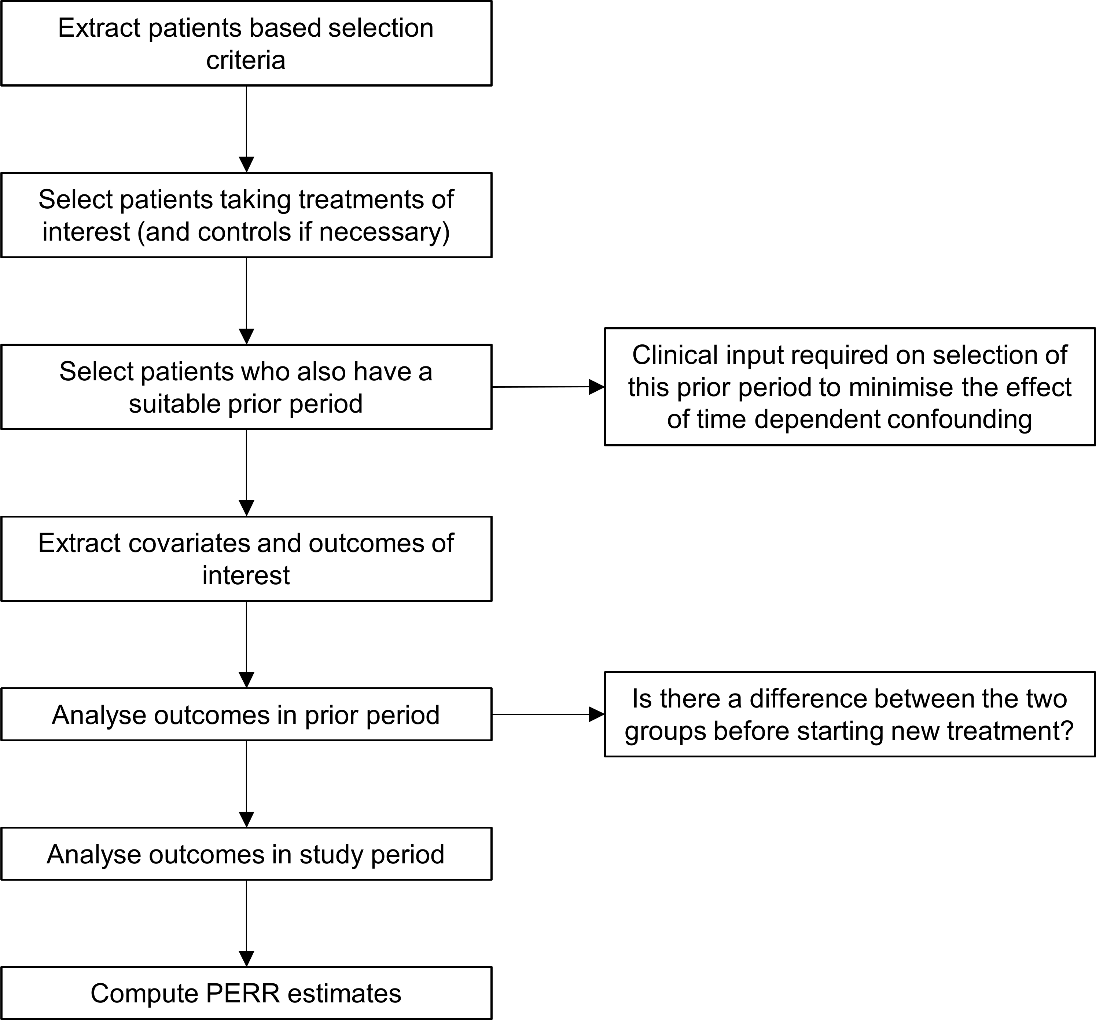
**

Fig B.1 General flow chart of data set construction and analysis stages

**C: Flowchart of dataset construction with case study data**

**
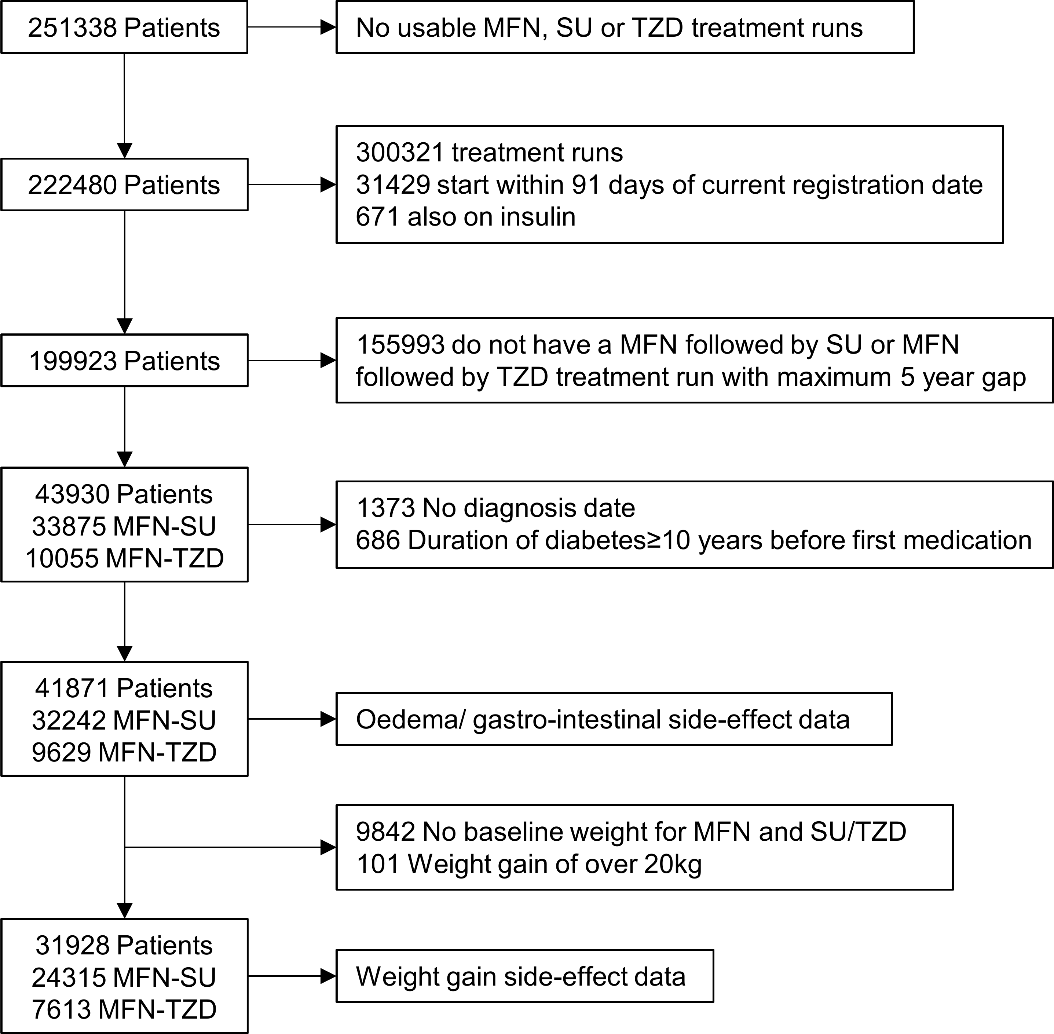
**

Fig C.1 Flowchart of dataset construction with case study data

**Appendix D: Time between start MFN and start SU/TZD**

**Oedema**

| Time between MFN and SU/TZD start | Prior period | Study period | PERR | PERR Pairwise | N |
| --- | --- | --- | --- | --- | --- |
| 3 years | 1.35 (1.1,1.66) | 2.15 (1.89,2.45) | 1.59 (1.26,2.01) | 1.34 (1,1.81) | 33174 |
| 4 years | 1.38 (1.14,1.66) | 2.17 (1.92,2.44) | 1.57 (1.28,1.94) | 1.42 (1.09,1.86) | 38356 |
| 5 years | 1.39 (1.17,1.66) | 2.12 (1.89,2.37) | 1.52 (1.24,1.87) | 1.39 (1.08,1.79) | 41871 |

Table D.1: Unadjusted models for side-effect Oedema with different periods of time between starting MFN and TZD/SU.

**Weight gain**

| Time between MFN and SU/TZD start | Prior period | Study period | PERR | PERR Pairwise | N |
| --- | --- | --- | --- | --- | --- |
| 3 years | 1 .0 (0.88,1.14) | 1.78 (1.66,1.91) | 1.77 (1.53,2.05) | 1.78 (1.49,2.12) | 25499 |
| 4 years | 0.97 (0.85,1.09) | 1.81 (1.69,1.93) | 1.87 (1.62,2.15) | 1.88 (1.59,2.22) | 29402 |
| 5 years | 1.01 (0.90,1.14) | 1.80 (1.69,1.92) | 1.78 (1.56,2.03) | 1.75 (1.49,2.05) | 32029 |

Table D.2: Unadjusted models for weight gain side-effect with different periods of time between starting MFN and TZD/SU.

**Gastrointestinal**

| Time between MFN and SU/TZD start | Prior period | Study period | PERR | PERR Pairwise | N |
| --- | --- | --- | --- | --- | --- |
| 3 years | 1.61 (1.44,1.79) | 1.70 (1.55,1.88) | 1.06 (0.92,1.22) | 0.97 (0.81,1.16) | 33174 |
| 4 years | 1.65 (1.49,1.81) | 1.61 (1.47,1.76) | 0.98 (0.87,1.1) | 0.88 (0.75,1.04) | 38356 |
| 5 years | 1.61 (1.47,1.77) | 1.63 (1.49,1.77) | 1.01 (0.90,1.13) | 0.93 (0.79,1.08) | 41871 |

Table D.3: Unadjusted models for gastrointestinal side-effect with different periods of time between starting MFN and TZD/SU.

**E: Model results – no adjustment for covariates**


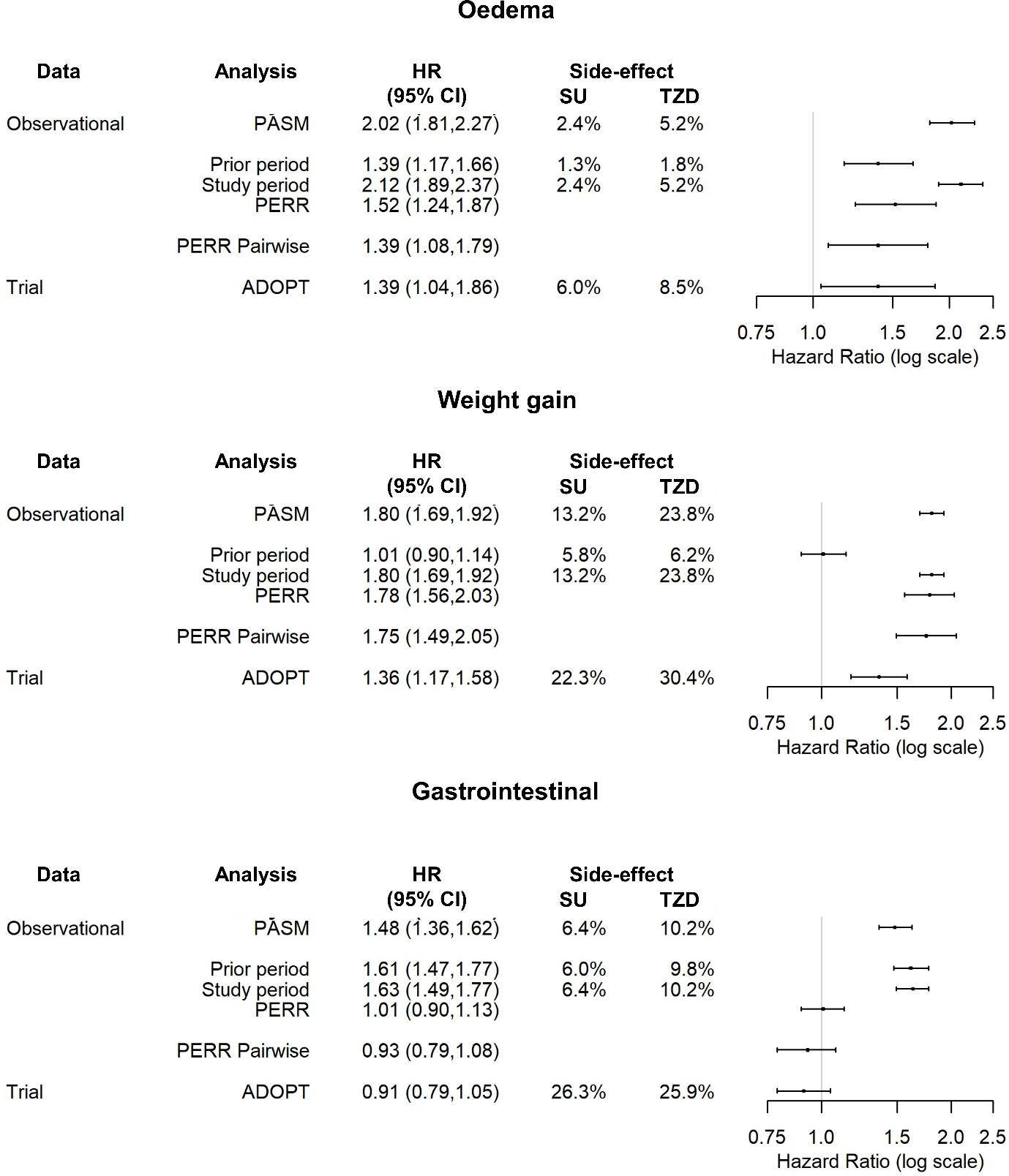


Figure E1: Observational data: N=32242 SU N=9629 TZD in Oedema and Gastrointestinal analyses, N=24327 SU N=7615 weight gain analysis. Column 4 shows percentage of patients who experience the side-effect in each period. An HR greater than 1 indicates a greater risk of side-effect in the TZD group relative to SU. An HR less than 1 indicates greater risk of a side-effect in the SU group. Where the confidence interval crosses 1 there is no significant difference between the two groups.

**F: Table of adjusted model results**

| Side-effect | Model | HR (95% CI) | Adjusted by | N | Percentage of patients experiencing side-effect in SU group | Percentage of patients experiencing side-effect in TZD group |
| --- | --- | --- | --- | --- | --- | --- |
| Oedema | Prior | 1.38 (1.13,1.68) | Gender, weight, age, calendar year of drug start | 22683 SU  7349 TZD | 1.4% | 1.9% |
|  | Study | 2.07 (1.81,2.37) | Gender, weight, age, HbA1c, calendar year of drug start, time difference between start MFN and secondline therapy |  | 2.4% | 5.3% |
|  | PERR | 1.50 (1.19,1.91) |  |  |  |  |
|  | Pairwise | 1.43 (1.10,1.83) | Duration of diabetes | 32242 SU  9629 TZD | 1.3% (prior period)  1.8% (study period) | 2.4% (prior period)  5.2% (study period) |
| Weight gain | Prior | 1.08 (0.92,1.25) | Gender, weight, HbA1c | 12972 SU  5120 TZD | 4.4% | 4.6% |
|  | Study | 1.70 (1.56,1.84) | Gender, weight, age, calendar year of drug start, HbA1c, adherence |  | 11.1% | 19.7% |
|  | PERR | 1.58 (1.33,1.90) |  |  |  |  |
|  | Pairwise | 1.52 (1.23,1.88) | Duration, HbA1c, adherence | 10807 SU  4468 TZD | 4.5% (prior period)  11.3% (study period) | 4.7% (prior period)  19.7% (study period) |
| Gastrointestinal |  |  |  |  |  |  |
|  | Prior | 1.47 (1.29, 1.68) | Gender, HbA1c, calendar year of drug start, adherence | 12796 SU  5142 TZD | 4.5% | 7.0% |
|  | Study | 1.60 (1.42,1.81) | Gender, HbA1c, calendar year of drug start, adherence |  | 5.2% | 9.1% |
|  | PERR | 1.09 (0.93,1.29) |  |  |  |  |
|  | Pairwise | 0.94 (0.80,1.10) | Duration of diabetes | 32242 SU  9629 TZD | 4.2% (prior period)  4.7% (study period) | 6.9% (prior period)  8.0 (study period) |

Table F1: N values differ due to missing values in covariates
